# Wearable sensors for Parkinson’s disease: which data are worth collecting for training symptom detection models

**DOI:** 10.1038/s41746-018-0071-z

**Published:** 2018-11-23

**Authors:** Luca Lonini, Andrew Dai, Nicholas Shawen, Tanya Simuni, Cynthia Poon, Leo Shimanovich, Margaret Daeschler, Roozbeh Ghaffari, John A. Rogers, Arun Jayaraman

**Affiliations:** 1 Max Nader Lab for Rehabilitation Technologies and Outcomes Research, Shirley Ryan AbilityLab, Chicago, IL 60611 USA; 2 Department of Physical Medicine and Rehabilitation, Northwestern University, Chicago, IL 60611 USA; 3 Department of Biomedical Engineering, Northwestern University, Evanston, IL 60208 USA; 4 Medical Scientist Training Program, Feinberg School of Medicine, Northwestern University, Chicago, IL 60611 USA; 5 Department of Neurology, Northwestern University, Chicago, IL 60611 USA; 6 The Michael J. Fox Foundation for Parkinson’s Research, New York, NY 10163 USA; 7 Center for Bio-Integrated Electronics, Departments of Materials Science and Engineering, Biomedical Engineering, Chemistry, Mechanical Engineering, Electrical Engineering and Computer Science, Neurological Surgery, Simpson Querrey Institute for Nano/Biotechnology, McCormick School of Engineering, Feinberg School of Medicine, Northwestern University, Evanston, IL 60208 USA; 8 Frederick Seitz Materials Research Laboratory, Department of Materials Science and Engineering, University of Illinois at Urbana-Champaign, Urbana, IL 61801 USA; 9 Department of Physical Therapy and Human Movement Sciences, Northwestern University, Chicago, IL 60611 USA

**Keywords:** Parkinson's disease, Rehabilitation, Diagnostic markers, Diagnostic markers

## Abstract

Machine learning algorithms that use data streams captured from soft wearable sensors have the potential to automatically detect PD symptoms and inform clinicians about the progression of disease. However, these algorithms must be trained with annotated data from clinical experts who can recognize symptoms, and collecting such data are costly. Understanding how many sensors and how much labeled data are required is key to successfully deploying these models outside of the clinic. Here we recorded movement data using 6 flexible wearable sensors in 20 individuals with PD over the course of multiple clinical assessments conducted on 1 day and repeated 2 weeks later. Participants performed 13 common tasks, such as walking or typing, and a clinician rated the severity of symptoms (bradykinesia and tremor). We then trained convolutional neural networks and statistical ensembles to detect whether a segment of movement showed signs of bradykinesia or tremor based on data from tasks performed by other individuals. Our results show that a single wearable sensor on the back of the hand is sufficient for detecting bradykinesia and tremor in the upper extremities, whereas using sensors on both sides does not improve performance. Increasing the amount of training data by adding other individuals can lead to improved performance, but repeating assessments with the same individuals—even at different medication states—does not substantially improve detection across days. Our results suggest that PD symptoms can be detected during a variety of activities and are best modeled by a dataset incorporating many individuals.

## Introduction

Parkinson’s disease (PD) is a neurological movement disorder that affects ~1% of people above 60 years of age in industrialized countries.^1,2^ Cardinal motor symptoms of PD that are responsive to levodopa therapy include tremors (particularly while at rest), rigidity, and slowness of movements (bradykinesia). These motor symptoms gradually worsen, hindering daily living and negatively impacting quality of life.^3,4^ Dopaminergic medications are used to alleviate some of these symptoms, but their effects tend to wear off more quickly as the disease progresses and affects wider domains. Some individuals also experience frequent changes in symptoms (‘OFF/ON’ state) or involuntary movements (dyskinesia) as a medication side effect,^5^ which in turn, forces individual adjustments in dosing. Tracking how motor symptoms and their response to medication change over time is crucial in quantifying the progression of PD for a given individual, in order to craft personalized treatment regimens.

The current gold standard of care to evaluate PD symptoms is through clinical examinations, whereby a trained clinician asks the patient to perform a series of standardized motor tasks (e.g. hand pronation-supination or heel tapping) while visually evaluating the quality of their movements and providing a symptom score. The most common rating scale to perform such evaluation is the Movement Disorder Society Unified Parkinson Disease’s Rating Scale (MDS-UPDRS).^6^ In addition, patients can be asked to keep a diary of their symptoms (e.g. Hauser diary,^7^ CAPSIT-PD,^8^ Parkinson’s Symptom Diary^9^). These methods are, however, limited by poor temporal resolution and low accuracy, respectively.^8^

Automatic evaluation of PD symptoms using wearable accelerometers, inertial, and electromyography sensors has been proposed as a way to overcome these limitations.^10^ Several studies have shown that it is possible to estimate MDS-UPDRS scores or detect motor symptoms using data from wearable sensors and machine learning models. Whereas signal processing has been used to manually design-specific features to identify symptoms,^11^ machine learning algorithms such as support vector machines or neural networks are increasingly used because of their ability to learn a classification model from the data and generalize to unseen scenarios. These models can be trained based on movement data collected from several individuals, as they perform standardized tasks in the clinic, as well as ground truth scores provided by clinicians.^12–15^ The data collection procedure often involves recording data from multiple subjects, wearing multiple sensors and undergoing repeated clinical assessments over several hours, so as to collect enough data on transitions between ‘OFF’ and ‘ON’ medication states. However, there is no consensus on the minimal data collection procedure, including number of sensors and sensor locations, or number of repeated clinical assessments and type of activities required to develop an accurate model while maximizing patient compliance.^16,17^ This information is critical towards facilitating the translation of this approach to real world symptom detection and monitoring in the clinic, as well as in the home and community.^18,19^

One of the main challenges for real world deployment is collecting sufficient labeled data to comprehensively model the varied presentation of motor symptoms during daily activities. In particular, symptoms may manifest differently across individuals and activities, and may also vary within the same individual over time.^20–23^ This renders deployment of symptom detection models in naturalistic environments challenging,^24,25^ and complicates the problem of generalizing symptom detection across individuals.^26^ As such, it is still unclear how many individuals and sensors data should be collected from, as well as whether data collected during several medication states is needed to train a system that can generalize symptom detection across days.

The objective of this study was to evaluate the relative value of several methods for increasing the amount of training data when developing machine learning models for PD symptom detection during activities representative of common daily tasks. We recorded movement data from 6 body-conforming flexible wearable sensors attached to the hands, arms, and thighs, and trained a machine learning classifier to detect the presence of tremor or bradykinesia in upper extremities, as individuals with PD performed a series of common daily activities and standard tasks used in clinical assessments. We trained statistical ensembles (random forest) and convolutional neural network (CNN) classifiers using data from a single sensor or multiple sensors, and evaluated the contribution of having sensors on both sides of the body. We then evaluated the effect of number of participants used to train the model on model performance. Finally, we evaluated whether incorporating training data from multiple clinical assessments rather than a single assessment improved the detection of these symptoms.

## Results

**Fig. 1:**
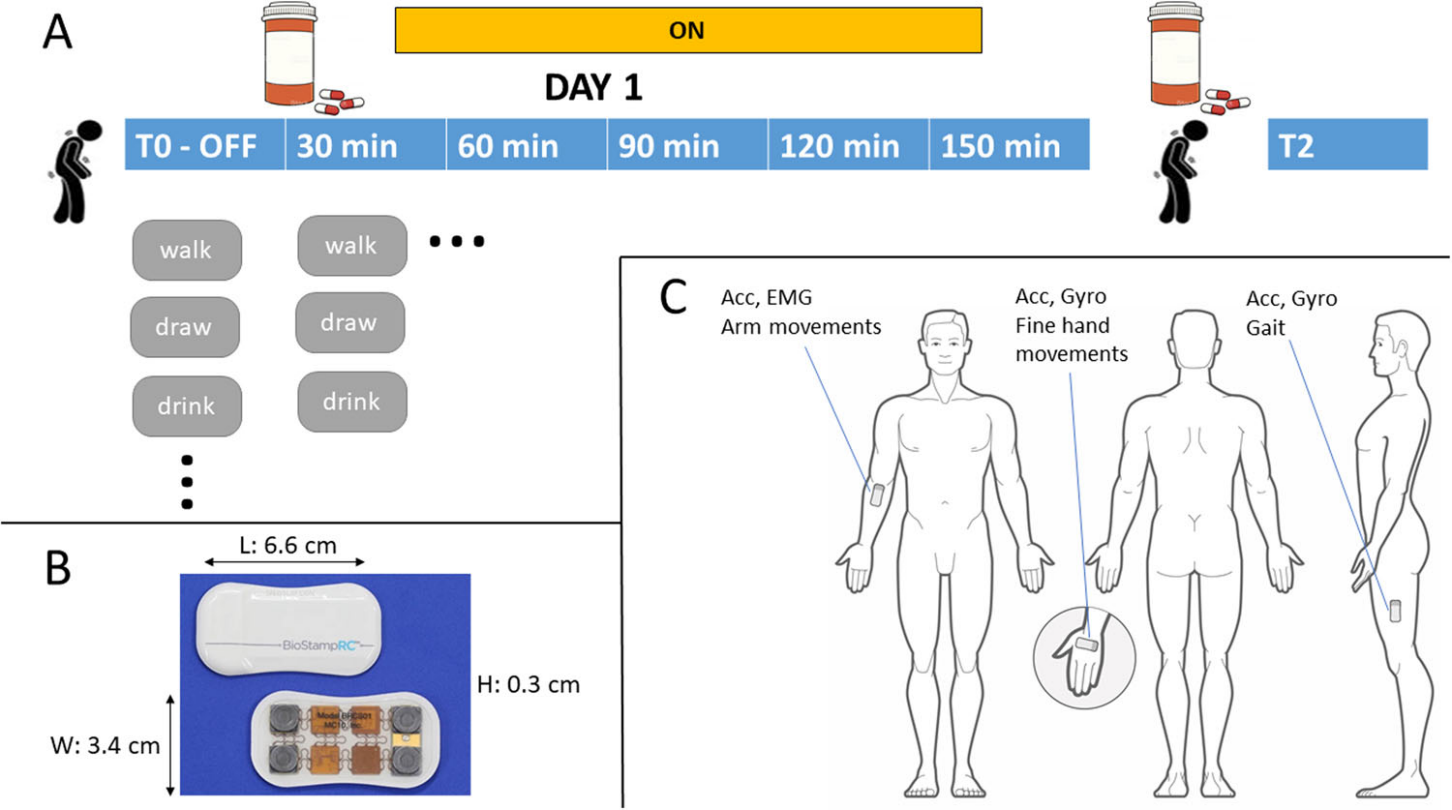
Data collection and sensor setup. **a** Individuals with PD underwent multiple clinical assessments spaced by 30 min during a first visit (day 1); assessments were done before and after each participant took their PD medication. A single follow-up assessment was performed about 2 weeks later during a second visit (day 2). During each assessment, participants performed a series of daily activities and standardized clinical tasks. **b** Overview of the MC10 BioStampRC senor; **c** Position of sensors on the body and sensor modalities; sensors were placed on both sides, although only one side is shown for clarity. Data were recorded from the accelerometer (acc) and gyroscope (gyro) sensors, or from the accelerometer and electromyography (EMG) sensor. Data from the EMG sensor was not used in the current study

Sensor data from 19 participants performing the repeated clinical assessments (Fig. 1a) were segmented into 5-second clips, yielding a total of 41,802 data clips. Demographic data of participants is reported in Table 1, whereas Table 2 contains the list of tasks used for the assessments. The mean proportion of task performances showing symptoms across participants were 48.5% (SD: 21.7%) for bradykinesia, 22% (SD: 24.4%) for tremor, and 8% (SD: 11.5%) for dyskinesias. The prevalence of tremor was substantially lower and more variable than that of bradykinesia, with one participant showing no manifestation of tremor at all. Because, the overall prevalence of dyskinesia in this dataset was low, and only 8 individuals showed dyskinesia during more than one task performance, we did not develop models for detection of dyskinesia. We trained separate random forest (RF) classifiers^27^ for the detection of each symptom (tremor or bradykinesia) from a set of features computed on the sensor data (Table 3). In addition, we compared the symptom detection models based on RF classifiers to those based on deep convolutional neural networks (CNNs). Details on the implementation are described in the Methods section.

**Table 1:** Participants’ demographics and associated clinical data

Participant ID	Sex	Age	Onset year	Diagnosis year	Fluctuator (Y/N)	Side predominantly affected at first assessment	MDS part III—day 1, time 0	MDS part III—day 1, time 60	MDS part III—day 2	Days between visits
1004	M	52	2011	2013	Y	Bilateral	31	30	14	11
1016	F	66	2016	2016	N	Bilateral	19	21	32	14
1018	M	58	2012	2015	N	Left	18	13	14	18
1019	F	36	2015	2015	N	Left	36	14	10	19
1020	F	58	2005	2005	N	Right	24	21	24	14
1024	M	70	2000	2000	Y	Left	42	18	19	22
1029	M	74	2009	2010	N	Left	42	32	21	13
1030	M	68	2010	2010	N	Left	18	NA	22	20
1032	M	70	2012	2012	N	Bilateral	28	12	26	16
1038	M	72	2007	2007	N	Right	30	25	20	69
1044	M	59	2013	2014	Y	Left	29	24	24	13
1046	F	69	2012	2014	N	Right	21	18	18	14
1047	M	52	2009	2010	Y	Right	18	9	6	20
1049	F	54	2006	2008	Y	Left	NA	24	24	13
1051	M	62	2013	2015	N	Left	14	6	11	49
1052	M	69	2007	2008	Y	Bilateral	31	15	NA	NA
1053	F	66	2014	2014	Y	Left	25	15	NA	NA
1054	F	65	2000	2002	Y	Right	44	15	NA	NA
1055	M	75	2006	2009	Y	Right	36	26	NA	NA
1056	M	72	2005	2006	Y	Left	46	61	NA	NA

**Table 2:** Tasks performed by participants during the visits for the assessment of PD symptoms

Task	Symptom detected	Type of task
Walking	Bradykinesia/Tremor	Functional
Walking while counting	Bradykinesia/Tremor	Functional
Finger to nose	Bradykinesia/Tremor	Clinical
Alternating hand movements	Bradykinesia/Tremor	Clinical
Sit to stand	Bradykinesia	Functional
Sitting	Tremor	Functional
Standing	Tremor	Functional
Drawing on paper	Bradykinesia/Tremor	Fine motor
Typing on a computer keyboard	Bradykinesia/Tremor	Fine motor
Nuts and bolts	Bradykinesia/Tremor	Fine motor
Pouring water from a bottle and drinking	Bradykinesia/Tremor	Gross motor
Organizing a set of folders	Bradykinesia/Tremor	Gross motor
Folding towels	Bradykinesia/Tremor	Gross motor

Tasks were divided into functional, fine motor, and gross motor groups

**Table 3:** Features computed on both the accelerometer and gyroscope data to train the symptom detection classifier

Feature	Feature dimension
Range (X,Y,Z)	3
Skew (X,Y,Z)	3
Kurtosis (X,Y,Z)	3
Cross-correlation peak (XY,XZ,YZ)	3
Cross-correlation lag (XY,XZ,YZ)	3
Dominant frequency (acceleration magnitude)	1
Relative magnitude	1
Moments of power spectral density (acceleration magnitude)	4
Moments of Jerk magnitude	4
Sample entropy (X,Y,Z)	3

### Effect of adding sensors

**Fig. 2:**
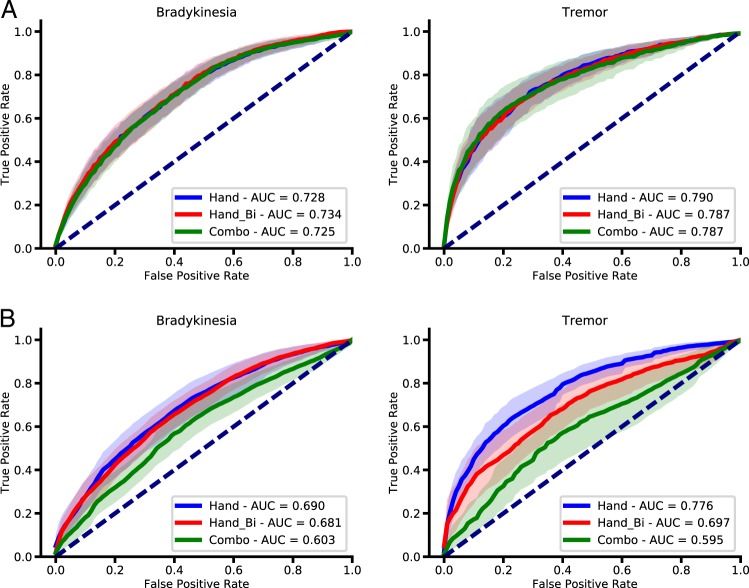
The effect of sensor location and number of sensors on model performance. AUROC curves for detection of bradykinesia and tremor using random forest population models **a** when trained on data from either a single hand (with dominant symptoms), both hands (Hand_Bi), or a combination of hand, forearm and thigh sensors unilaterally (Combo). Corresponding AUROC curves from CNN population models **b** for detection of bradykinesia and tremor. Using a combination of sensors did not yield any advantage to only using hand sensors. Solid lines indicate mean AUROC and shaded areas represent 95% confidence intervals

Random forest models trained on hand data yielded the highest mean AUROC for both the detection of bradykinesia (0.73, 95% CI: 0.68–0.77) and tremor (0.79, 95% CI: 0.74–0.84) across all activities (Fig. 2a). Using data from sensors on both hands to detect symptoms on the impaired side did not significantly improve performance (bradykinesia: 0.73, CI: 0.69–0.78; paired *t*-test, *t* = −0.74, *p* = 0.47; tremor: 0.79, CI: 0.74–0.84; paired *t*-test, *t* = 0.35, *p* = 0.73). Similarly, using data from hand, arm, and thigh sensors together did not confer a significant advantage relative to using one hand sensor only for bradykinesia (0.73, 95% CI: 0.68–0.77; paired *t*-test, *t* = 0.54, *p* = 0.59) or for tremor (0.79, 95% CI: 0.72–0.86; *t* = 0.19, *p* = 0.85).

Using a CNN-based approach showed a similar result (Fig. 2b bradykinesia—single hand: 0.69, CI: 0.63–0.75; hands bilateral: 0.68, CI: 0.63–0.73, *p* = 0.72; tremor—single hand: 0.78, CI: 0.73–0.82; hands bilateral: 0.70, CI: 0.62–0.77, *p* = 0.06); using data from all sensors significantly degraded model performance (bradykinesia: 0.60, CI: 0.54–0.66, *p* = 0.014; tremor: 0.60, CI: 0.50–0.69, *p* = 0.001), suggesting overfitting of the data. Therefore, detection of symptoms in the upper extremities across all activities was best achieved using one sensor placed on the hand with the dominant symptoms. All subsequent analyses use only data from such hand sensor.

### Symptom detection across activities

**Fig. 3:**
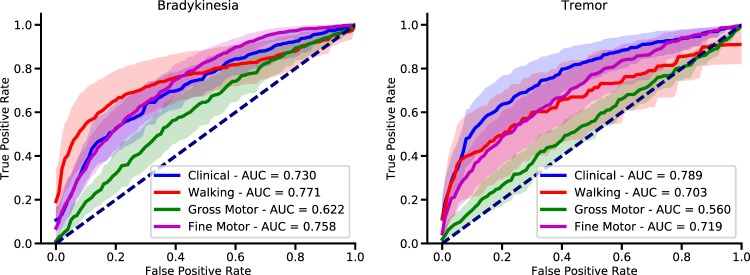
Symptom detection across activities. AUROC curves for bradykinesia and tremor detection using population models (random forest), split by groups of activities. Symptoms were detected equally well during both clinical structured tasks (e.g. finger to nose movements) and most of daily functional activities (walking and fine motor tasks, e.g. typing on a keyboard). The lowest AUROC was during the execution of gross motor tasks

Random forest models were able to detect bradykinesia and tremor during both clinical structured activities (finger to nose and alternating hand movements), and activities of daily living (Fig. 3). Bradykinesia detection during fine motor tasks and walking yielded the highest mean AUROC across participants (walking: 0.77, CI: 0.67–0.87; fine motor: 0.76, CI: 0.70–0.81); detection accuracy was not significantly different from that achieved on clinical structured tasks (0.73, CI: 0.66–0.80, *p*> = 0.50). In contrast, detection during gross motor tasks had the lowest AUROC and was significantly lower compared to that on clinical tasks (0.62; CI: 0.54–0.70, *p* = 0.04). Tremor was most accurately detected from clinical tasks (0.79; CI: 0.71–0.88), though mean AUROC here was not significantly higher than AUROC for walking (0.70; CI: 0.53–0.88; *p* = 0.25) or fine motor tasks (0.72; CI: 0.62–0.81, *p* = 0.23). Again, detection during the execution of gross motor tasks was significantly worse than during clinical tasks (mean AUROC: 0.56; 95% CI: 0.49–0.63; *p* < 0.001).

Overall, AUROC values for CNN models for detection of bradykinesia and tremor were comparable to those of random forest models. Detection performance was highly variable across subjects, and as such there was no significant difference in mean AUROC values (*p*> = 0.26) (see Supplementary Material). Interestingly, detection of bradykinesia from walking achieved better results than for all other tasks (*p* = 0.02). This suggests that it is possible to detect symptoms from some daily activities and structured clinical assessments with comparable accuracies.

### Effect of number of training subjects

To evaluate the effect on accuracy of number of subjects in the training dataset, we trained random forest models on data from an increasing number of participants (from 3 to 18). At each iteration, a model was trained on a random subset of participants and tested on the left-out ones.

**Fig. 4:**
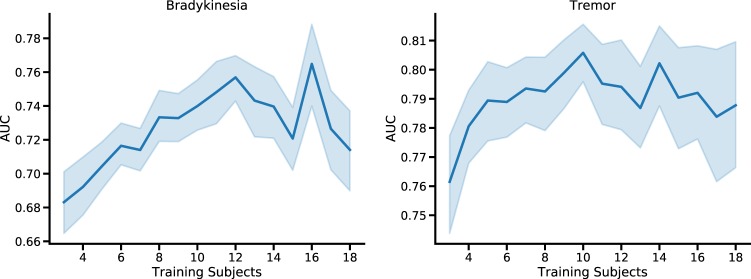
Comparing performance of population models with different number of individuals in the training pool. Plots showing the trend in expected AUROC for bradykinesia and tremor detection when testing on a new individual, when using population models with varying numbers of individuals in the training pool. Shaded areas mark 95% confidence interval on the mean

Models for tremor detection showed a noticeable improvement with added training subjects (Fig. 4). The mean AUC when evaluating data from a new subject improved from 0.76 (CI: 0.74–0.78) for a training set using data from only three individuals to 0.80 (CI: 0.79–0.82, *p* < 0.001) for a training set using data from 10 individuals. However, improvement seemed to plateau, showing limited improvement beyond that point. Similarly, for bradykinesia detection, increased training pool size also correlated with increases in expected AUC (three subjects: 0.68, CI: 0.66–0.70; 12 subjects, 0.76, CI: 0.74–0.77, *p* < 0.001), and plateaued around a similar number of training subjects.

Due to the large number of trainable parameters, CNNs require larger datasets than random forest models to prevent overfitting, and therefore were not included in this analysis.

### Effect of number of training sessions

**Fig. 5:**
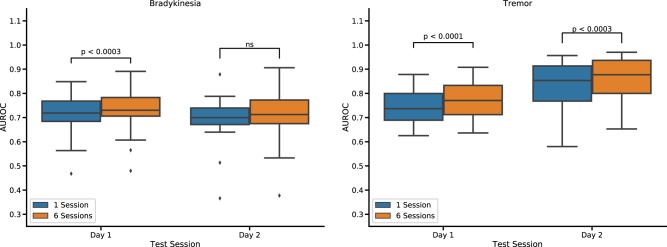
The effect of using training data from multiple assessments (session) on model performance. Boxplots show the distribution of AUROC across participants for population models trained on data from day 1 and tested on either day 1 or day 2. Adding data from multiple sessions improves the AUROC for bradykinesia detection on day 1. However, no significant changes in AUROC occur when models are tested on data from a different day (day 2). A similar pattern is observed for detection of tremor, although a modest improvement was observed on day 2

We analyzed whether training models on data from multiple clinical assessments (sessions) performed on day 1 improved their performance at detecting symptoms on the same day or on a different day (day 2). Random forest models for detection of bradykinesia benefited from adding training data collected over multiple sessions, relative to models trained on a single session, when tested on data from the same day (Fig. 5). However, the improvement was modest (mean AUROC change: 0.02; *p* < 0.001), and was not significant when models were tested on data from a subsequent visit (mean AUROC change population: 0.02, *p* = 0.053).

A similar pattern was observed for detection of tremor (day 1: mean AUROC change population: 0.03, *p* < 0.001), although a small but significant improvement was observed also on day 2 when multiple sessions data were added (day 2: mean AUROC change population 2: 0.03, *p* < 0.001). As above, CNNs were not included in this analysis as they tend to underperform with smaller datasets. Therefore, collecting data from repeated assessments does not substantially help generalization across days.

## Discussion

This study aimed to investigate how to efficiently collect wearable sensor data for detection of bradykinesia and tremor in the upper extremities. To that end, we evaluated the effects on model performance of number and location of wearable sensors used, types of tasks performed by participants, and number of data collection sessions performed. In addition, we evaluated how many individuals are needed to reach optimal model performance. We used random forest classifiers, based on pre-engineered features, and compared them to convolutional neural networks trained on raw sensors data.

Overall, CNN-based models showed comparable mean AUROC values to those of classifiers based on random forest. This suggests that the networks can learn features for detection of PD symptoms directly from the raw sensor data, without the need of engineering a feature set. Although CNNs have the potential to learn representations of the data that can distinguish between different activities or contexts (i.e. walking vs. gross vs. fine motor tasks), we did not observe any substantial improvement relative to random forest models for the detection of symptoms. Future work should explore alternative architectures for the CNN, including LSTM models^28^ to see whether any improvement in generalization can be obtained.

Another novelty of our study was the use of soft wearable sensors,^29^ which adhere to the skin and are able to conform to its deformation during movements. This allows greater flexibility in sensor placement for collecting data from any part of the body, and with minimal burden for the wearer. Our results suggest that a single motion sensing device placed on the hand is sufficient to detect symptoms of tremor or bradykinesia in the upper extremities. This is in agreement with a previous study evaluating the minimum number of traditional wearables to estimate bradykinesia.^16^

Combining data from sensors on both sides of the body did not aid detection on the impaired side, for bradykinesia and tremor. Other studies have evaluated the contribution of using non-movement sensing modalities, such as EMG, though it is unclear how necessary these modalities are for symptom detection.^30^ It is also possible that the optimal combination of sensor locations and modalities may be dependent on the types of tasks that are analyzed, but this study was focused on the evaluation of upper limb symptoms. It remains to be seen whether soft sensors provide an advantage over traditional wearables in terms of improved symptom detection during daily activities.

We also found that fine motor tasks and walking are suitable activities for automated evaluation of PD symptoms in the upper limbs using a single-hand sensor. Detection of bradykinesia and tremor from fine motor tasks or walking had comparable accuracy to detection from tasks used for clinical evaluation of these symptoms (e.g. finger to nose movements). Symptom detection during gait has previously been focused primarily on freezing of gait, rather than bradykinesia or tremor.^11,31–34^ Other studies have shown that motor symptoms can be quantified by prompting users to perform gait and finger tapping tests at home.^35,36^ Our results suggest that fine motor tasks and walking may also be useful targets for symptom monitoring during naturalistic behaviors at home.

We observed that increasing the number of subjects in the training dataset improved model performance, even if accuracy tended to decrease when the model was trained on data from more than 10 individuals. This may be partially due to the high variability in manifestation of PD symptoms, particularly bradykinesia, across individuals. Therefore, a smaller amount of appropriately targeted data may lead to more accurate models than a larger pool of more general data. In the field of activity recognition, some studies have proposed methods for developing “semi-personalized” models.^37^ The best method for doing so in the context of symptom detection and scoring in Parkinson’s disease should be explored in future studies.

Collecting data from repeated assessments significantly improved symptom detection, although to a limited extent. Incorporating data from ‘ON’ and ‘OFF’ medication states, as well as transition states may aid symptom detection, but this effect seems to be attenuated in population models. Interestingly, bradykinesia detection on a separate visit (day 2) was not improved by increasing the number of data collection sessions used for training. The difference in model performance could be explained by changes in the behavior of participants,^38^ or in the medication state of participants at the subsequent visit. Sensors were removed between visits and replaced at the second visit, which may also explain this observation. Therefore, generalizing symptom detection to a different day does not seem to be aided by the use of data from additional assessments, even when altering the medication state.

The cost-benefit ratio of training data collection needs to be considered when developing symptom detection models for PD, especially given the need for a trained clinician to evaluate the presence and severity of symptoms. It may be important to target-specific types of activity, as symptom detection is improved during fine motor tasks and walking relative to gross upper extremity activity. Our findings also indicate that increasing the number of body sensor devices, the number of subjects whose data is used for training models, or the number of data collection sessions per individual does not necessarily translate into an improved accuracy for detection of bradykinesia and tremor. Unique strategies for leveraging the value of extremely large datasets (deep learning), or for personalizing detection models (transfer learning) are likely to be important directions for continued improvement of accuracy in motor symptom detection outside of the clinic.

### Limitations

Because of the limited number of individuals in this study who exhibited symptoms of dyskinesia, this study did not examine models for dyskinesia detection. The low number of dyskinesia events may be in part associated to restricting the monitoring to upper extremity symptoms only. Dyskinesia is a significant side effect of levodopa use, and is another important target of symptom monitoring in patients with PD. A study with a larger number of individuals may be necessary to capture sufficient instances of dyskinesia and to perform a similar analysis for detection of this important symptom.

Our study is limited in exploring longitudinal model performance due to having available data for only two distinct visits. Additionally, not all participants were able to complete the second visit, leading to reduced statistical power in that analysis. Also, data used here were collected in a supervised clinical environment, and thus is not perfectly representative of real-world behaviors. However, the tasks used here were intended to approximate some typical daily activities that patients might perform. Accelerometer data collected from a smartwatch during an extended period of time outside the clinic, as performed in the parent CIS-PD study, may be able to address this limitation and will be explored in a future analysis.

Another limitation of our study was the fact that we only had one rater for scoring the standardized motor assessments. Therefore, we could not estimate the inter-rater variability, and thus the uncertainty of the target scores provided to the model. Furthermore, whereas our aim was to evaluate the relative contribution of different data sources to the accuracy of detection models, our dataset was still limited in size to draw general conclusions. Future studies should examine these aspects with a larger and broader dataset in terms of subjects and monitoring periods.

## Methods

### Study design

Twenty individuals (13 males; mean age: 63.35 ± 9.63 y) diagnosed with PD and a Hoehn and Yahr stage of 2 provided written consent and participated in the study, which took place at Northwestern Memorial Hospital (Chicago, IL, USA) and was approved by the Northwestern University Institutional Review Board (IRB #: STU00203796). Ten participants were considered as “motor fluctuators”, meaning that they were ‘OFF’ for an average of 2 h daily, as determined by the clinician interview and confirmed by the modified Hauser diary; the remaining 10 participants tended to experience stable symptoms before and after taking medications (Table 1). One participant had a deep brain stimulation (DBS) device implanted. This analysis was a sub-study (Wireless Adhesive Sensor Sub-Study) of a larger multi-center study sponsored by the Michael J. Fox Foundation and named “Clinician Input Study on Parkinson Disease” (CIS-PD), which aimed at developing objective biomarkers to monitor Parkinson’s symptoms based on wearable sensors data.

In order to capture changes in symptom severity over time the study included two sets of experimental visits (day 1 and day 2), performed in the clinic on 2 different days, spaced about 2–3 weeks apart. The day 1 visit consisted of 6 repeated assessments (sessions), spaced by at least 30 min apart (Fig. 1a). Participants were instructed to come to the clinic without taking any PD medication for at least 12 h prior to the visit, so as to start the assessment in the ‘OFF’ medication state. After completing the first session, they were instructed to take their PD medications, in order to assess their symptoms as they transition into the ‘ON’ medication state. During each session, participants performed 13 different motor tasks (standardized motor assessments, Table 2), some of which resembled daily activities with others being standard clinical tasks used to elicit symptoms, e.g. finger to nose and alternating hand movements. A trained clinician rated the severity of tremor, bradykinesia and dyskinesia in the upper extremities for the left and right side on a scale from 0 to 4 during the execution of each task (Table 2). The day 2 visit consisted of a single session and was meant to introduce longitudinal variability and characterize participants’ symptoms on a completely different day and during their regular medication schedule. Only the first 15 participants performed the day 2 assessment. In addition to the aforementioned activities, an MDS-UPDRS (Part III) motor assessment was also performed during session 1 and 3 (time 0 and 60 minutes) on day 1, and during the visit on day 2. The total MDS-UPDRS score for each subject are reported in Table 1. Sensor data from one of the participants (1020) were not used as not all of their clinical assessments were rated by the trained neurologist.

### Sensor setup

Study participants were instrumented with 6 BioStampRC sensors (MC10 Inc., Lexington, MA, USA), which are flexible wearable sensors that can be attached directly to the skin and include a tri-axial accelerometer, a gyroscope, and a single lead (2 electrodes) analog voltage sensor (Fig. 1b). A single BioStampRC can record data from up to 2 sensors simultaneously. Here, sensors were placed on the dorsal part of each hand, on each thigh proximal of the femur epicondyle, and on each forearm on top of the flexor carpi radialis (Fig. 1c). All sensors were set to record data from the tri-axial accelerometer (sampling rate: 62.5 Hz; range: ±4G) and from the gyroscope (sampling rate: 62.5 Hz; range:±1000 °/s), except for the forearm sensors which recorded the acceleration and the electromyography (EMG) signal from the left and right flexor carpi radialis muscles (1000 Hz). Data from each sensor were initially stored on the local memory (32 MB), then transmitted to a tablet via Bluetooth, and finally uploaded to the MC10 cloud server. EMG data were not used in the current analysis.

### Data analysis and preprocessing

Data processing and analysis was performed using Python 3.5. Sensor data for each participant was downloaded and organized according to side and sensor modality, as well as activity and session based on the timestamp information recorded during data collection using the BioStampRC app. Data from sensors on the left (right) side was matched with clinical scores for bradykinesia, tremor and dyskinesia for the corresponding side. All sensor data (accelerometer and gyroscope) were segmented into 5-second clips with 50% overlap, and data from each clip was filtered using a 4th order Butterworth filter. The length of the time window was chosen according to previous work^12,13^ and considering the average time scale of activities (e.g. opening a water bottle, pouring, and drinking), Accelerometer data were filtered with a high-pass filter (0.5 Hz) to remove the effect of limb orientation; in addition, a low-pass filter with cutoff at 3 Hz was applied to the accelerometer and gyroscope data for bradykinesia detection. Such a choice has been found to help detection of symptoms in previous studies.^12^ Accelerometer and gyroscope data from each clip were matched with their corresponding metadata (patient ID, side, activity, session, clinical score). A total of 41,802 clips were generated from this process. Out of these, 41% had bradykinesia and 22% tremor. There were not enough examples of dyskinesia (8%), and therefore these data were not used to train a dyskinesia detection model.

### Symptom detection models

We trained 2 random forest (RF) classifiers:^27^ one for detecting whether a data clip had bradykinesia, and the other for tremor. Therefore, clinical scores >0 for each clip were assigned a value of 1 (symptom present). Each classifier inputted a vector of 56 features (Table 3), which were calculated on both the accelerometer and gyroscope data from each clip. These features capture movement properties related to speed and frequency and were successfully used in prior PD studies.^12^ We used RF because of the low number of hyperparameters to be tuned and its ability to reduce overfitting. The number of trees was set to 50 and was optimized using the out-of-bag error method.

In addition, we trained two classification models based on deep convolutional neural networks (CNN). CNN-based models can perform end-to-end learning of symptom detection from the raw sensors data, and therefore does not need the development of a set of features to encode a data clip. Features representing the relationship between sensor data and symptoms are learned from the raw data and encoded in the weights across the network layers. CNNs have been used in a variety of tasks, such as image classification, speech recognition, and natural language processing (NLP), where they tend to outperform traditional approaches based on feature engineering.^39^ Deep networks have also been used to classify wearable sensor data for activity recognition^28^ and recently symptom detection in Parkinson’s^14,31^, where they proved to be at least as effective as traditional methods based on feature engineering.^39^ Each CNN (Fig S1) consisted of the following layers: 2 convolutional layers (kernel sizes = 32 and 16 samples) with 16 and 32 rectified linear units (ReLU), respectively, each followed by a max-pooling layer (pool-size = 4 and 6 units). The last two layers are 2 dense layers with 32 neurons each, also with ReLU activation functions. Dense layers used dropout,^40^ such that a fixed proportion (0.5) of units were ‘shut down’ during training to reduce overfitting. The output layer used a softmax function for the classification, with as many neurons as classes (2), which output the probability of the input clip showing a symptom (bradykinesia/tremor). The total number of trainable parameters for each model was 24,722. Further details on the CNN models are provided in supplementary material.

We trained population-based models, i.e, models trained on data from other subjects to detect bradykinesia and tremor in a new subject. Area under the receiver operating characteristic curve (AUROC) was used to assess model performance. Mean AUROC across subjects was computed using a leave-one-subject-out cross validation for population models. Statistical comparisons were performed using *t*-test for either paired or independent samples.

### Code availability

The code used to process and analyze the findings of this publication are available in a GitHub repository, [https://github.com/Luke3D/CIS_PD-NDM/].

## Electronic supplementary material

Supplemental Material

## Data Availability

The dataset used to support the findings of this publication are available from the Michael J. Fox Foundation but restrictions apply to the availability of these data, which were used under license for this study. The Michael J. Fox Foundation plans to release the dataset used in this publication alongside a significant, additional portion of related PD data from a separate smartwatch as part of a community analysis in the larger CIS-PD study timeline. Data are, however, available from the authors upon reasonable request and with permission from the Michael J. Fox Foundation.

## Electronic supplementary material

**Supplementary information** accompanies the paper on the *npj Digital Medicine* website (10.1038/s41746-018-0071-z).
