# Supplementary material for: Wearable sensors for Parkinson’s disease: which data are worth collecting for training symptom detection models
Source: NPJ Digit Med. 2018 Nov 23;1:64. doi: 10.1038/s41746-018-0071-z (PMC6550186; doi:10.1038/s41746-018-0071-z)
Supplement: Supplementary file 1 — Supplemental Material [file 41746_2018_71_MOESM1_ESM.pdf]

# Supplementary Material

## Wearable sensors for Parkinson's disease: which data is worth collecting for training symptom detection models

Luca Lonini[*1,2], Andrew Dai[*1,3], Nicholas Shawen[*1,4], Tanya Simuni[5], Cynthia Poon[5], Leo Shimanovich[5], Margaret Daeschler[6], Roozbeh Ghaffari[7], John A. Rogers[7,8], Arun Jayaraman[1,2,9]

[*]These authors contributed equally to this work

Michael J. Fox Foundation for Parkinson's Research Clinician Input Study (CIS-PD) Wireless Adhesive Sensor Sub-Study

[1]Max Nader Lab for Rehabilitation Technologies and Outcomes Research, Shirley Ryan AbilityLab, Chicago, IL 60611, USA

[2]Department of Physical Medicine and Rehabilitation, Northwestern University, Chicago, IL 60611, USA

[3]Department of Biomedical Engineering, Northwestern University, Evanston, IL 60208, USA

[4]Medical Scientist Training Program, Feinberg School of Medicine, Northwestern University, Chicago, IL 60611, USA

[5]Department of Neurology, Northwestern University, Chicago, IL 60611, USA

[6]The Michael J. Fox Foundation for Parkinson's Research, New York, NY 10163, USA

[7]Center for Bio-Integrated Electronics, Departments of Materials Science and Engineering, Biomedical Engineering, Chemistry, Mechanical Engineering, Electrical Engineering and Computer Science, Neurological Surgery, Simpson Querrey Institute for Nano/Biotechnology, McCormick School of Engineering, Feinberg School of Medicine, Northwestern University, Evanston, IL 60208, USA

[8]Frederick Seitz Materials Research Laboratory, Department of Materials Science and Engineering, University of Illinois at Urbana-Champaign, Urbana, IL 61801, USA

[9]Department of Physical Therapy and Human Movement Sciences, Northwestern University, Chicago, IL 60611, USA

**Corresponding Author**:

Arun Jayaraman, PT, PhD
Max Nader Lab for Rehabilitation Technologies and Outcomes
Shirley Ryan AbilityLab
355 E. Erie St.
11th Floor, Room 1401
Chicago, IL 60611, USA
ajayaraman@sralab.org
+1 (312) 238-6875

# Contents

**1. Models based on deep neural networks**
1.1. Symptom detection across activities

## 1. Models based on deep neural networks

Here we report in more detail the structure of the convolutional neural network (CNN) and the parameters used for training. In addition, we include the results of the analysis of symptom detection across activities.

Each CNN model consists of the following layers (Fig. S1): 2 convolutional layers (kernel sizes $f$= 32 and 16 samples, with stride $s$=1) with 16 and 32 rectified linear units (ReLU) respectively, each followed by a max-pooling layer (pool-size $f$ =4 and 6 units, with strides $s$=2 and 4). The last 2 layers are 2 dense (or fully connected) layers with 32 neurons each, also with ReLU activation functions. Dense layers used dropout[1], such that a fixed proportion (0.5) of units were 'shut down' during training to reduce overfitting. The output layer used a Softmax function for the classification, with as many neurons as classes (2), which outputs the probability of the input clip showing a symptom (bradykinesia/tremor). The total number of trainable parameters for each model was 24,722.

The network parameters (weights) are updated so to minimize the cross-entropy loss function between predicted and reference labels, by using the Adam optimizer[2], a stochastic gradient-descent optimization algorithm. We set the parameters to their default values as recommended in the paper (learning rate of 0.001, beta_1=0.9, beta_2=0.999). At each training iteration, we randomly draw mini-batches of 1024 data points (clips), until all data points from the training dataset are drawn, which represents an epoch. The model is trained for a maximum of 30 epochs, using early stopping: as soon as the error on the validation batches is not decreasing for 5 consecutive epochs, learning is stopped. We performed a random transformation of the input batches, by simulating a random rotation of the sensor in their plane. The angle of rotation is drawn uniformly at random between -5 and 5 degrees. This process injects artificial noise into the data to simulate the process of positioning sensors to slightly different orientation in every trial, and therefore increasing generalization[3].

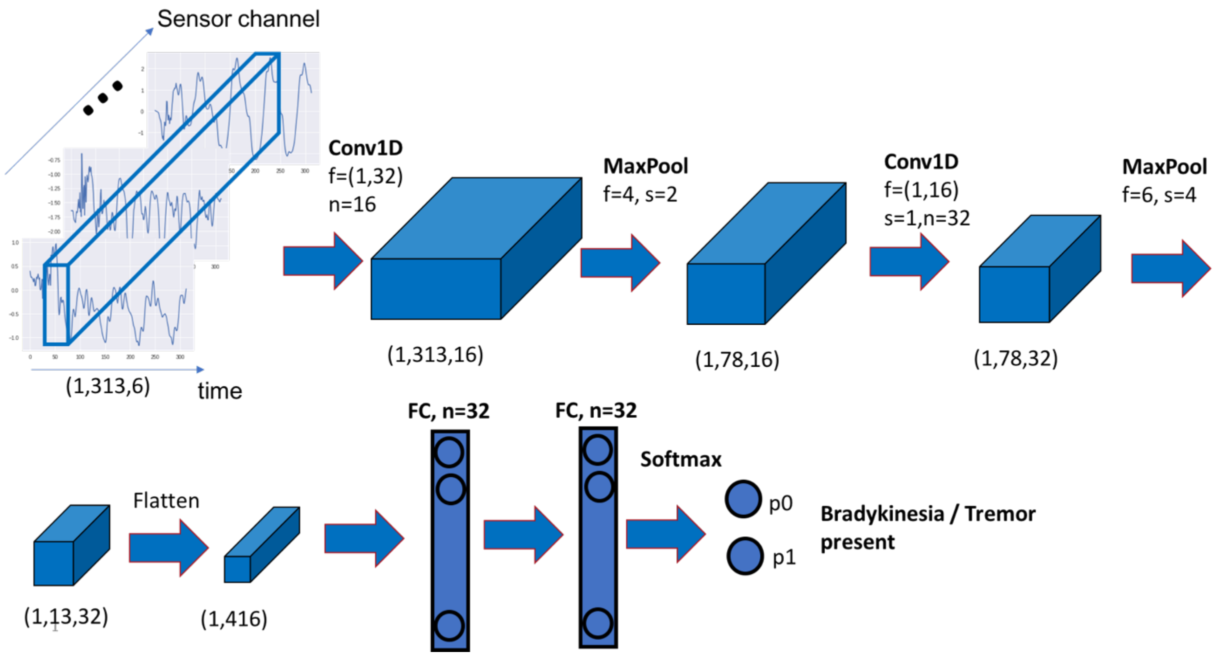


Fig. S1 CNN model for symptom detection: the network inputs the raw sensors data and outputs the probability that a symptom is present (p1) or not (p0 = 1-p1). Each convolutional layer is depicted with a box, where the 3rd dimension corresponds to the number of filters in that layer; *f* denotes the kernel size, *s* is the stride and *n* the number of filters in the layer. An input data clip is a tensor of dimension (1, 313, N_channels). FC stands for fully connected (or dense) layers.

We trained population models based on CNNs, and evaluated them against the effect of sensor location and on detection of symptoms across different groups of activities.

### 1.1 Symptom detection across activities

Detection of bradykinesia during walking tasks yielded the highest mean AUROC (0.78, CI:0.69-0.88), while detection during clinical tasks was significantly worse (0.64, CI:0.57-0.72; p=0.02). Detection during gross and fine motor tasks were not significantly different from detection during clinical tasks (Gross: 0.64, CI:0.58-0.70; Fine: 0.66, CI:0.58-0.75; p>=0.65). Detection of tremor yielded comparable mean values of AUROC across clinical (0.72, CI:0.62-0.82), walking (0.71, CI:0.42-1.00) and fine motor tasks (0.74, CI:0.66-0.82); mean AUROC was lower during gross motor tasks (0.62, CI:0.56-0.68) (Fig.S2). However, detection performance was highly variable across subjects, and as such there was no significant difference in mean AUROC values (p>=0.26). Therefore, CNN-based population models were best at detecting bradykinesia from walking tasks, while did not show any difference across activities for tremor detection.

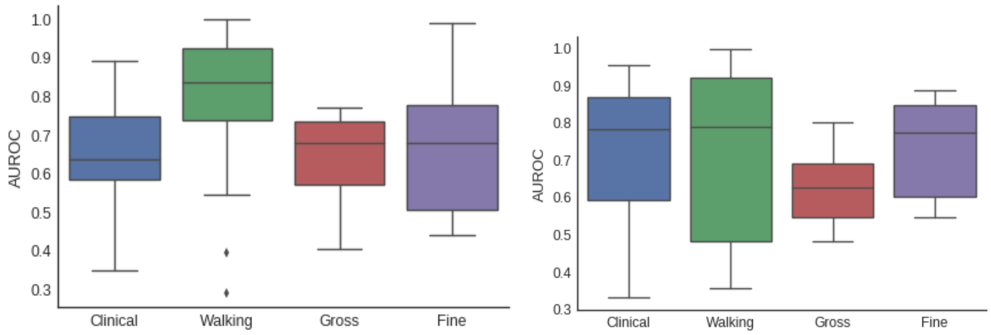

Fig. S2: AUROC distribution across subjects for CNN-based models trained on detection of bradykinesia (left) and tremor (right), split by group of tasks.

## References

1. Srivastava, N., Hinton, G., Krizhevsky, A., Sutskever, I. & Salakhutdinov, R. Dropout: A Simple Way to Prevent Neural Networks from Overfitting. *J. Mach. Learn. Res.* **15,** 1929–1958 (2014).
2. Kingma, D. P. & Ba, J. L. ADAM: A METHOD FOR STOCHASTIC OPTIMIZATION. In *International Conference on Learning Representations (ICLR)* (Vol. 5).
3. Um, T. T. *et al.* Data Augmentation of Wearable Sensor Data for Parkinson's Disease Monitoring using Convolutional Neural Networks. in *Proceedings of the 19th ACM International Conference on Multimodal Interaction* (2017). doi:10.1145/3136755.3136817
